# Multicenter development of a PET-based risk assessment tool for product-specific outcome prediction in large B-cell lymphoma patients undergoing CAR T-cell therapy

**DOI:** 10.1007/s00259-023-06554-0

**Published:** 2023-12-20

**Authors:** Conrad-Amadeus Voltin, Andrea Paccagnella, Michael Winkelmann, Jan-Michel Heger, Beatrice Casadei, Laura Beckmann, Ken Herrmann, Franziska J. Dekorsy, Nadine Kutsch, Peter Borchmann, Stefano Fanti, Wolfgang G. Kunz, Marion Subklewe, Carsten Kobe, Pier Luigi Zinzani, Matthias Stelljes, Katrin S. Roth, Alexander Drzezga, Richard Noppeney, Kambiz Rahbar, H. Christian Reinhardt, Bastian von Tresckow, Robert Seifert, Jörn C. Albring, Viktoria Blumenberg, Andrea Farolfi, Sarah Flossdorf, Philipp Gödel, Christine Hanoun

**Affiliations:** 1 Department of Nuclear Medicine, Faculty of Medicine and University Hospital Cologne, University of Cologne, Cologne, Germany; 2 Department of Experimental, Diagnostic, and Specialty Medicine (DIMES), University of Bologna, Bologna, Italy; 3 Department of Radiology, University Hospital Munich, Ludwig Maximilian University Munich, Munich, Germany; 4 Department of Internal Medicine I, Center for Integrated Oncology Aachen–Bonn–Cologne–Düsseldorf (CIO ABCD), Faculty of Medicine and University Hospital Cologne, University of Cologne, Cologne, Germany; 5 Cologne Lymphoma Working Group (CLWG), Cologne, Germany; 6 ‘L. e A. Seràgnoli’ Institute of Hematology, Scientific Institute for Research, Hospitalization, and Healthcare (IRCCS) ‘Azienda Ospedaliero-Universitaria Di Bologna’, University of Bologna, Bologna, Italy; 7 Department of Nuclear Medicine, University Hospital Essen, University of Duisburg-Essen, Essen, Germany; 8 German Cancer Consortium (DKTK) Partner Site Essen/Düsseldorf, Essen, Germany; 9 Department of Nuclear Medicine, University Hospital Munich, Ludwig Maximilian University Munich, Munich, Germany; 10 Division of Nuclear Medicine, Scientific Institute for Research, Hospitalization, and Healthcare (IRCCS) ‘Azienda Ospedaliero-Universitaria Di Bologna’, University of Bologna, Bologna, Italy; 11 Department of Medicine III, Comprehensive Cancer Center Munich (CCCM), University Hospital Munich, Ludwig Maximilian University Munich, Munich, Germany; 12 Laboratory for Translational Cancer Immunology, Gene Center Munich, Ludwig Maximilian University Munich, Munich, Germany; 13 German Cancer Consortium (DKTK) and Bavarian Center for Cancer Research (BZKF) Partner Site Munich, Munich, Germany; 14 Department of Medicine A–Hematology, Oncology, and Pneumology, West German Cancer Center (WTZ) Network Partner Site, University Hospital Münster, University of Münster, Münster, Germany; 15 Department of Hematology and Stem Cell Transplantation, West German Cancer Center (WTZ), University Hospital Essen, University of Duisburg-Essen, Essen, Germany; 16 Department of Nuclear Medicine, University Hospital Münster, University of Münster, Münster, Germany; 17 Institute for Medical Informatics, Biometry, and Epidemiology, University Hospital Essen, University of Duisburg-Essen, Essen, Germany

**Keywords:** Large B-cell lymphoma, CAR T-cell therapy, Risk factors, Extra-nodal involvement, MTV

## Abstract

**Purpose:**

The emergence of chimeric antigen receptor (CAR) T-cell therapy fundamentally changed the management of individuals with relapsed and refractory large B-cell lymphoma (LBCL). However, real-world data have shown divergent outcomes for the approved products. The present study therefore set out to evaluate potential risk factors in a larger cohort.

**Methods:**

Our analysis set included 88 patients, treated in four German university hospitals and one Italian center, who had undergone 2-[^18^F]fluoro-2-deoxy-D-glucose positron emission tomography (PET) before CAR T-cell therapy with tisagenlecleucel or axicabtagene ciloleucel. We first determined the predictive value of conventional risk factors, treatment lines, and response to bridging therapy for progression-free survival (PFS) through forward selection based on Cox regression. In a second step, the additive potential of two common PET parameters was assessed. Their optimal dichotomizing thresholds were calculated individually for each CAR T-cell product.

**Results:**

Extra-nodal involvement emerged as the most relevant of the conventional tumor and patient characteristics. Moreover, we found that inclusion of metabolic tumor volume (MTV) further improves outcome prediction. The hazard ratio for a PFS event was 1.68 per unit increase of our proposed risk score (95% confidence interval [1.20, 2.35], *P* = 0.003), which comprised both extra-nodal disease and lymphoma burden. While the most suitable MTV cut-off among patients receiving tisagenlecleucel was 11 mL, a markedly higher threshold of 259 mL showed optimal predictive performance in those undergoing axicabtagene ciloleucel treatment.

**Conclusion:**

Our analysis demonstrates that the presence of more than one extra-nodal lesion and higher MTV in LBCL are associated with inferior outcome after CAR T-cell treatment. Based on an assessment tool including these two factors, patients can be assigned to one of three risk groups. Importantly, as shown by our study, metabolic tumor burden might facilitate CAR T-cell product selection and reflect the individual need for bridging therapy.

**Supplementary Information:**

The online version contains supplementary material available at 10.1007/s00259-023-06554-0.

## Introduction

Chimeric antigen receptor (CAR) T‐cell therapy provides a new option for patients with various lymphoma subtypes. Tisagenlecleucel and axicabtagene ciloleucel are anti-CD19 products that were approved in relapsed or refractory large B-cell lymphoma (LBCL) based on results of the JULIET and ZUMA-1 trials [1, 2]. While the design heterogeneity of these studies, mainly with respect to inclusion criteria and bridging therapy, precludes direct comparison, recently published real-world data show considerable progression-free survival (PFS) as well as overall survival (OS) differences between the two constructs [3, 4].

The importance of individualized treatment planning is already widely appreciated, since CAR T-cell therapy can cause clinically relevant side effects and requires complex patient management [5, 6]. However, a specific risk model on which to base treatment decisions has not yet been established. CAR T-cell product selection and indications for tumor debulking are contingent upon local standards of care and hence inconsistent. The available data suggest that, among other factors, lymphoma burden and response to bridging therapy may influence outcomes after CAR T-cell infusion [7, 8]. Moreover, intrinsic tumor factors and characteristics of the cells administered such as dose or kinetics have been discussed [9–11].

Positron emission tomography (PET) with 2-[^18^F]fluoro-2-deoxy-D-glucose ([^18^F]FDG) is a diagnostic modality routinely used for the staging of patients undergoing CAR T-cell therapy. Recently, retrospective studies including our own have demonstrated that quantitative image evaluation by parameters like metabolic tumor volume (MTV) may be helpful in pretreatment assessment [12–15]. We therefore initiated this multicenter analysis to find a risk assessment tool for the specific context of CAR T-cell therapy based on conventional disease characteristics, patient factors, and PET metrics, taking into account potential differences between products. Additionally, the role of lymphoma burden before treatment in the development of toxicities was examined.

## Patients and methods

### Data collection

Our study cohort included patients with relapsed or refractory biopsy-proven LBCLs who had undergone CAR T-cell therapy through January 31, 2021, and met the following criteria:

1. PET examination performed within 30 days before infusion of tisagenlecleucel or axicabtagene ciloleucel
2. No systemic cytoreductive treatment after imaging besides a lymphodepleting regimen of fludarabine and cyclophosphamide
3. Absence of lesions only captured by another diagnostic modality or classified as non-measurable due to high physiologic [^18^F]FDG uptake in the surrounding tissue

After institutional ethics committee approval, the four participating German university hospitals and single Italian center identified 88 individuals who qualified for analysis. The study sites were asked to provide information about clinical stage, extra-nodal disease sites, patient age, Eastern Cooperative Oncology Group status, lactate dehydrogenase (LDH) levels, C-reactive protein values, therapy lines, and response status after bridging treatment. Product selection was based on the approved indications and local availability. All individuals or their representatives gave written informed consent for CAR T-cell therapy and the respective staging procedures. Forty-seven of the patients enrolled were also examined in a smaller analysis recently published elsewhere [14].

### PET scanning and quantitative image analysis

Imaging was performed at the academic medical centers that participated in our study, using Biograph mCT, Biograph mMR, Biograph Vision (Siemens Healthcare GmbH, Erlangen, Germany); Discovery MI (GE HealthCare, Milwaukee, WI); and Gemini TF (Koninklijke Philips N.V., Amsterdam, The Netherlands) PET systems. As part of clinical protocol, scans were acquired according to institutional standards.

MTV was calculated semi-automatically with a standardized uptake value (SUV) threshold of 4.0 in ACCURATE (PETRA consortium, Amsterdam, The Netherlands), LIFEx (Laboratory of Translational Imaging in Oncology, Orsay, France) [16], MIM Encore (MIM Software, Inc., Cleveland, OH), or syngo.via (Siemens Healthcare GmbH, Erlangen, Germany), depending on reader preferences. Recent studies indicate a satisfactory level of agreement between the software tools used [14, 17, 18]. The absolute cut-off chosen has been shown previously to achieve the highest delineation success rates in lymphoma patients [19]. Furthermore, we measured the maximum SUV (SUV_max_) as an additional PET parameter.

### Statistical evaluation and development of a predictive model

Main patient outcomes evaluated by our study were PFS, defined as time to imaging-detected progression, relapse, or any-cause death, and OS, which was calculated taking only deaths into account. We determined 1-year survival rates through Kaplan–Meier analysis and used Cox regression with likelihood ratio tests to assess differences between risk groups. Median follow-up time was calculated by the reverse Kaplan–Meier method. Moreover, hazard ratios of the identified risk factors and corresponding 95% Wald’s confidence limits were established.

Our model for prediction of PFS was developed by multivariable Cox regression based on forward selection, using the Akaike’s information criterion (AIC). All conventional disease parameters and basic patient characteristics collected were taken into account as factors potentially affecting outcome. In a second step, we assessed whether MTV and SUV_max_ could improve the generated model. The respective optimal threshold was determined individually for each CAR T-cell product based on AIC with the condition that at least 15% of patients should be allocated to a risk group. Factors which proved predictive were analyzed through univariable Cox regression and in the context of our final model. Additionally, our study evaluated whether grade of cytokine release (CRS) or immune effector cell-associated neurotoxicity syndrome (ICANS), assessed using the American Society for Blood and Marrow Transplantation consensus scoring system [20], correlates with metabolic tumor burden based on Pearson’s coefficients separately for both CAR T-cell constructs. An excessive response of immune cells following infusion primarily causes fever in CRS. It may also lead to hypotension, capillary leak, and end-organ dysfunction. CAR T-cell-related ICANS is a pathologic process involving the central nervous system, which can induce aphasia, impairment of consciousness or cognitive skills, motor weakness, seizures, and cerebral edema.

To examine potential differences in baseline characteristics between the two treatment subgroups receiving tisagenlecleucel and axicabtagene ciloleucel, we performed Mann–Whitney *U* as well as Fisher’s exact tests. All analyses were conducted with SAS software (SAS Institute, Inc., Cary, NC), treating the study sites as random effects in Cox models.

## Results

### Baseline characteristics of the study cohort

The median patient age was 59 years, with a male-to-female ratio of 1.67 (Table 1). A majority of them were treated for relapsed or refractory diffuse LBCL (*n* = 67, 76.1%), and elevated LDH was the most common established risk factor (*n* = 61, 69.3%). While 62 individuals underwent CAR T-cell therapy with tisagenlecleucel (70.5%), 26 received axicabtagene ciloleucel (29.5%). Though, generally, the baseline characteristics of our two treatment groups did not significantly differ, there were a few exceptions: first, a higher metabolic tumor burden (*P* = 0.024) but less frequent need for bridging therapy of patients who received axicabtagene ciloleucel (*P* < 0.001) and, second, differences in histologic subtype distribution (*P* = 0.023). A markedly lower proportion of individuals undergoing treatment with tisagenlecleucel had primary mediastinal B-cell lymphoma.

**Table 1:** Patient and disease characteristics in the two treatment subgroups

	Tisagenlecleucel (*n* = 62)	Axicabtagene ciloleucel (*n* = 26)	*P*
Age, y			
Median	58	61	0.621
Range	19–82	19–73	
Sex			
Female	25 (40.3)	8 (30.8)	0.474
Male	37 (59.7)	18 (69.2)	
Lymphoma subtype			
Diffuse LBCL	49 (79)	18 (69.2)	0.023
Transformed follicular lymphoma	9 (14.5)	2 (7.7)	
Primary mediastinal B-cell lymphoma	2 (3.2)	6 (23.1)	
High-grade B-cell lymphoma	2 (3.2)	0 (0)	
IPI factors			
Age > 60 years	29 (46.8)	14 (53.9)	0.642
Ann Arbor stage III or IV	40 (64.5)	19 (73.1)	0.469
More than one extra-nodal lesion	33 (53.2)	19 (73.1)	0.1
ECOG performance status ≥ 2	17 (27.4)	8 (30.8)	0.798
Elevated LDH	46 (74.2)	15 (57.7)	0.137
IPI score			
1–2	30 (48.4)	14 (53.9)	0.816
3–5	32 (51.6)	12 (46.2)	
Bulky disease*			
Yes	11 (17.7)	6 (23.1)	0.566
No	51 (82.3)	20 (76.9)	
Treatment lines			
Median	3	3	0.095
Range	2–12	1–7	
Prior stem-cell transplantation			
Autologous	20 (32.3)	7 (26.9)	0.801
Allogeneic	1 (1.6)	0 (0)	
Bridging therapy			
Yes	62 (100)	19 (73.1)	< 0.001
No	0 (0)	7 (26.9)	
Response to bridging treatment			
Complete remission	6 (9.7)	0 (0)	0.082
Partial response	20 (32.3)	2 (10.5)	
Stable disease	7 (11.3)	4 (21.1)	
Progressive disease	29 (46.8)	13 (68.4)	
MTV			
Median	55	202	0.024
Range	0–1831	5–3646	
SUV_max_			
Median	19.3	19.8	0.661
Range	2.4–54.8	5.7–38.8	

Data are presented as *n* (%) of patients unless otherwise indicated

*ECOG* Eastern Cooperative Oncology Group, *IPI* International Prognostic Index, *LBCL* large B-cell lymphoma, *LDH* lactate dehydrogenase, *MTV* metabolic tumor volume, *SUV*_*max*_ maximum standardized uptake value

^*^Presence of a lesion measuring ≥ 7.5 cm in at least one axis

### Optimal PET parameter thresholds and risk categorization

The most suitable MTV and SUV_max_ cut-offs to predict PFS were 11 mL and 9.7, respectively, for patients treated with tisagenlecleucel. In contrast, markedly higher thresholds of 259 mL and 15.1 provided optimal predictive power among those receiving axicabtagene ciloleucel.

**Fig. 1:**
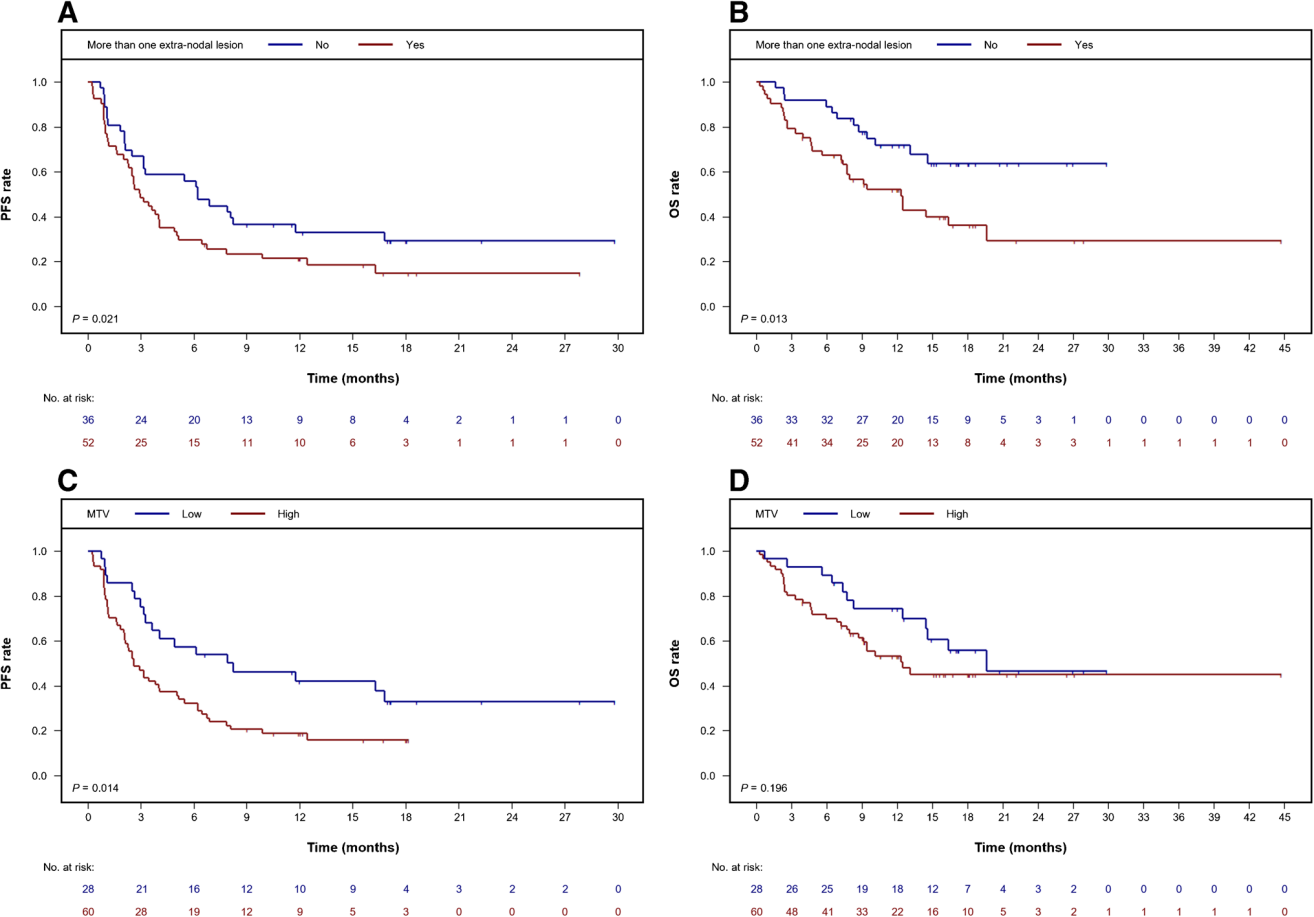
Kaplan–Meier estimates for predictive factors identified. Impact of extra-nodal disease (**A**, **B**) and MTV (**C**, **D**) on survival outcomes. MTV, metabolic tumor volume; OS, overall survival; PFS, progression-free survival

Extra-nodal disease emerged as the most relevant of the conventional tumor and patient characteristics (Fig. 1A, B) with a hazard ratio (HR) of 1.83 (95% confidence interval (CI) [1.10, 3.04], *P* = 0.021). No other factors considered for the first risk modeling step were of any added value in our study cohort. However, with a univariable HR of 2.04 (95% CI [1.16, 3.58], *P* = 0.014), metabolic tumor burden was identified as a PET parameter that can further improve PFS prediction and formed the developed assessment tool’s second category (Fig. 1C, D). Accordingly, patients were assigned to one of three risk groups based on a numerical scale ranging between 0 and 2.

**Fig. 2:**
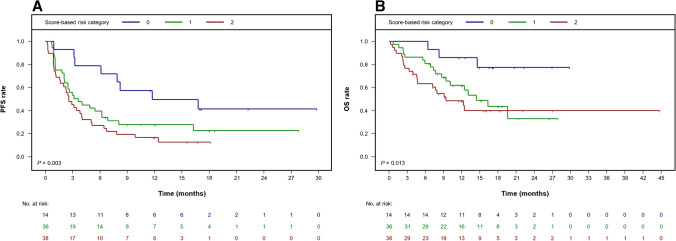
Risk assessment with a two-factor score including the presence of more than one extra-nodal lesion and MTV. Kaplan–Meier curves for PFS (**A**) and OS (**B**) based on individual status. MTV, metabolic tumor volume; OS, overall survival; PFS, progression-free survival

In our study, which had a 17-month median follow-up, individuals with no more than one extra-nodal lesion plus smaller MTV achieved 1-year PFS and OS probabilities of 49.0% (95% CI [28.4, 84.5]) and 85.7% (95% CI [69.2, 100]), respectively. The HR for a PFS event per unit increase of our sum score comprising these two risk factors was 1.68 (95% CI [1.20, 2.35]; *P* = 0.003; Fig. 2). Accordingly, 1-year PFS and OS rates in patients who had extra-nodal disease as well as a higher metabolic lymphoma burden were significantly lower at 15.8% (95% CI [7.6, 32.9]) and 48.2% (95% CI [34.3, 67.7]), respectively.

### Correlation between tumor burden and grades of toxicity

**Fig. 3:**
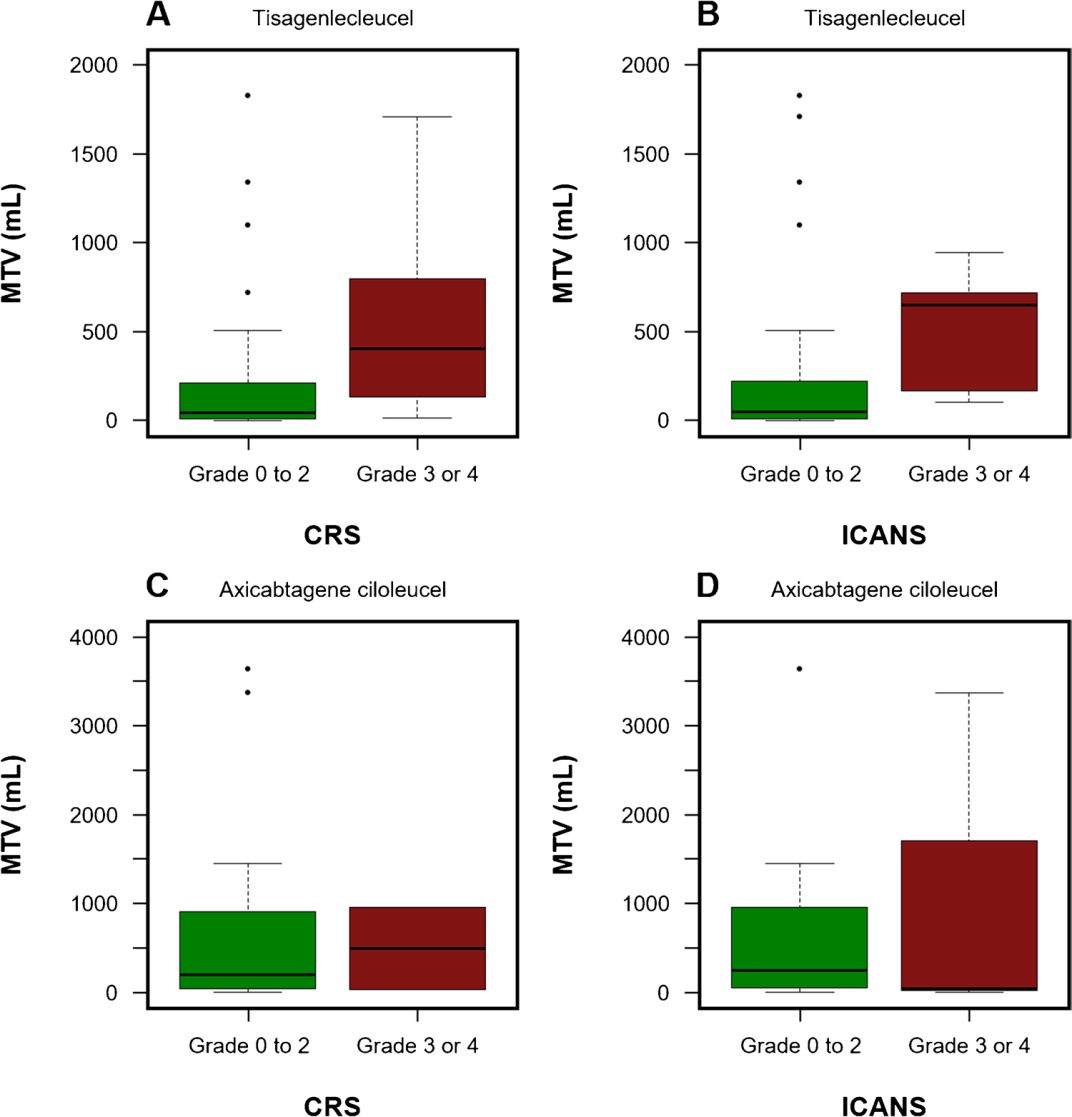
Box plots comparing the distribution of MTV in patients with negligible to moderate versus more severe CRS (**A**, **C**) and ICANS (**B**, **D**) by CAR T-cell product. CAR, chimeric antigen receptor; CRS, cytokine release syndrome; ICANS, immune effector cell-associated neurotoxicity syndrome; MTV, metabolic tumor volume

We observed a weak correlation of metabolically active tumor volume with CRS (*r* = 0.359; *P* = 0.004) and ICANS grade (*r* = 0.298; *P* = 0.019) for individuals receiving tisagenlecleucel. No relevant associations of MTV to CRS (*r* = 0.242; *P* = 0.235) or ICANS severity (*r* = 0.059; *P* = 0.776) after CAR T-cell treatment were found in axicabtagene ciloleucel-treated patients. The lymphoma burden distribution of individuals with negligible or moderate versus more severe toxicity is illustrated by Fig. 3.

## Discussion

We here present a product-specific risk assessment tool for CAR T-cell therapy in LBCL that includes extra-nodal disease reflecting lesion dissemination and pretreatment MTV as a measure of tumor load. The PET parameter cut-offs were chosen taking into account potential differences between tisagenlecleucel and axicabtagene ciloleucel. Our two-factor score accurately identified patients at risk of shorter PFS and OS after CAR T-cell treatment. In those with more than one extra-nodal lesion and elevated MTV, the rate of progression or death was three times higher than for individuals without.

Extra-nodal spread was the most relevant of conventional patient characteristics and basic disease parameters, a finding consistent to previous studies [13, 14]. Shorter survival in these individuals could be explained by the observed hindrance of T-cell migration into non-lymphatic tissues [21]. Hence, optimization of the approved products and tailored bridging strategies have the potential to improve therapy outcomes.

Interestingly, extra-nodal disease and MTV compared favorably with several established risk factors. This may be explained by the circumstance that Ann Arbor stage and LDH are only surrogates of tumor burden. Using large clinical trial data sets, Mikhaeel et al. developed a risk score based on metabolic tumor burden, patient age, and clinical stage, which predicted the treatment response of diffuse LBCL patients undergoing first-line therapy more accurately than the International Prognostic Index [18, 22]. In the setting of CAR T-cell treatment, that novel risk assessment tool was found to be predictive for PFS but not OS [23].

To date, there is no randomized study comparing the efficacy of tisagenlecleucel and axicabtagene ciloleucel. However, real-world data have recently shown significant differences regarding survival rates [3, 4]. Our analysis indicates that axicabtagene ciloleucel may be particularly beneficial in patients with high lymphoma load, as the optimal MTV threshold of tisagenlecleucel was markedly lower. Metabolic tumor burden would thus seem useful for the selection of both CAR T-cell product and bridging therapy. Of note, unlike others [12, 24], we did not use the MTV median as a threshold to identify individuals at risk, but determined specific cut-offs based on model selection methods, providing increased accuracy. Several studies indicate that effective debulking is most important in high-tumor volume patients, while those who have low MTV should be protected from chemo- or radiotherapy-associated toxicity. The decrease of metabolic tumor burden between leukapheresis and CAR T-cell infusion significantly correlated with PFS in a study by Sesques et al. [24]. Surprisingly, we did not find any predictive value of response status after bridging treatment. This might be explained by the fact that disease stratification based on Lugano criteria [25] is less precise than the exact measurement of volume changes.

While other authors have reported an association between MTV and CRS development [26, 27], the present study showed only weak correlations of lymphoma load with CAR T-cell-specific adverse events. However, it is important to note that the number of higher-grade toxicities was limited in our cohort. Furthermore, management of these potentially life-threatening side effects has significantly improved over time, presumably through the use of tocilizumab and corticosteroids at an increasingly early stage [28, 29].

Implementation of standardized measuring methods and automated workflows will be essential if MTV or other PET-derived biomarkers are to become available in routine clinical practice. Radiomics may also play a relevant role within the management of patients undergoing CAR T-cell therapy [30]. Recent developments suggest that more convenient tools such as plug-ins for commercial imaging software can be expected soon. The deep-learning algorithm presented by Jemaa et al. is just one of several with sufficient accuracy in lymphoma [31]. We ourselves used the absolute SUV threshold of 4.0 for semi-automatic tumor burden calculation. This cut-off is currently the most promising candidate, since it was found to be the least influenced by image reconstruction and choice of segmentation tool [17, 32].

One potential weakness of our study lies in the retrospective data collection. Nevertheless, the findings appear generalizable to a broad spectrum of patients, as individuals from five university centers and two European countries were enrolled. Another strength was the inclusion of two different CAR T-cell products. However, it should be noted that the treatment groups were not matched for all baseline characteristics. Although cautious interpretation is needed due to the limited number of patients who received either tisagenlecleucel or axicabtagene ciloleucel, respectively, we believe that both risk factors identified will become increasingly important (Supplementary Fig. 1). Thus, there remains a particular need for reliable data on the efficacy of different bridging strategies in cases with higher tumor load. Moreover, prospective studies should be carried out to confirm the prognostic value of extra-nodal disease as well as MTV and refine treatment recommendations not only for tisagenlecleucel or axicabtagene ciloleucel but also other CAR T-cell products like lisocabtagene maraleucel [33].

## Conclusions

Our study demonstrates that extra-nodal disease and higher MTV identified by [^18^F]FDG PET are associated with a significantly worse outcome following CAR T-cell treatment in LBCL. Using an assessment tool, which includes these two factors, patients can be assigned to one of three risk groups. Moreover, as shown by the present analysis, metabolic tumor burden might be a valuable parameter for selection of the optimal CAR T-cell product and bridging therapy.

### Supplementary Information

Below is the link to the electronic supplementary material.

Supplementary file1 (DOCX 359 KB)

## Data Availability

All data generated and analyzed during our study are available from the corresponding author on reasonable request.
