# Supplementary material for: Multicenter development of a PET-based risk assessment tool for product-specific outcome prediction in large B-cell lymphoma patients undergoing CAR T-cell therapy
Source: Eur J Nucl Med Mol Imaging. 2023 Dec 20;51(5):1361–70. doi: 10.1007/s00259-023-06554-0 (PMC10957657; doi:10.1007/s00259-023-06554-0)
Supplement: Supplementary file 1 — Supplementary file1 (DOCX 359 KB) [file 259_2023_6554_MOESM1_ESM.docx]

*Original article – Supplementary material*

**Multicenter development of a PET-based risk assessment tool for product-specific outcome prediction in large B-cell lymphoma patients undergoing CAR T-cell therapy**

Conrad-Amadeus Voltin,^1^ Andrea Paccagnella,^2^ Michael Winkelmann,^3^ Jan-Michel Heger,^4,5^ Beatrice Casadei,^2,6^ Laura Beckmann,^4^ Ken Herrmann,^7,8^ Franziska J. Dekorsy,^9^ Nadine Kutsch,^4,5^ Peter Borchmann,^4,5^ Stefano Fanti,^2,10^ Wolfgang G. Kunz,^3^ Marion Subklewe,^11,12,13^ Carsten Kobe,^1^ Pier Luigi Zinzani,^2,6^ Matthias Stelljes,^14^ Katrin S. Roth,^1^ Alexander Drzezga,^1^ Richard Noppeney,^8,15^ Kambiz Rahbar,^16^ H. Christian Reinhardt,^8,15^ Bastian von Tresckow,^8,15^ Robert Seifert,^7,8,16^ Jörn C. Albring,^14^ Viktoria Blumenberg,^11,12,13^ Andrea Farolfi,^10^ Sarah Flossdorf,^17^ Philipp Gödel,^4,5^ and Christine Hanoun^8,15^

^1^ Department of Nuclear Medicine, Faculty of Medicine and University Hospital Cologne, University of Cologne, Cologne, Germany

^2^ Department of Experimental, Diagnostic, and Specialty Medicine (DIMES), University of Bologna, Bologna, Italy

^3^ Department of Radiology, University Hospital Munich, Ludwig Maximilian University Munich, Munich, Germany

^4^ Department of Internal Medicine I, Center for Integrated Oncology Aachen – Bonn – Cologne – Düsseldorf (CIO ABCD), Faculty of Medicine and University Hospital Cologne, University of Cologne, Cologne, Germany

^5^ Cologne Lymphoma Working Group (CLWG), Cologne, Germany

^6^ 'L. e A. Seràgnoli' Institute of Hematology, Scientific Institute for Research, Hospitalization, and Healthcare (IRCCS) 'Azienda Ospedaliero-Universitaria di Bologna', University of Bologna, Bologna, Italy

^7^ Department of Nuclear Medicine, University Hospital Essen, University of Duisburg-Essen, Essen, Germany

^8^ German Cancer Consortium (DKTK) Partner Site Essen/Düsseldorf, Essen, Germany

^9^ Department of Nuclear Medicine, University Hospital Munich, Ludwig Maximilian University Munich, Munich, Germany

^10^ Division of Nuclear Medicine, Scientific Institute for Research, Hospitalization, and Healthcare (IRCCS) 'Azienda Ospedaliero-Universitaria di Bologna', University of Bologna, Bologna, Italy

^11^ Department of Medicine III, Comprehensive Cancer Center Munich (CCCM), University Hospital Munich, Ludwig Maximilian University Munich, Munich, Germany

^12^ Laboratory for Translational Cancer Immunology, Gene Center Munich, Ludwig Maximilian University Munich, Munich, Germany

^13^ German Cancer Consortium (DKTK) and Bavarian Center for Cancer Research (BZKF) Partner Site Munich, Munich, Germany

^14^ Department of Medicine A – Hematology, Oncology, and Pneumology, West German Cancer Center (WTZ) Network Partner Site, University Hospital Münster, University of Münster, Münster, Germany

^15^ Department of Hematology and Stem Cell Transplantation, West German Cancer Center (WTZ), University Hospital Essen, University of Duisburg-Essen, Essen, Germany

^16^ Department of Nuclear Medicine, University Hospital Münster, University of Münster, Münster, Germany

^17^ Institute for Medical Informatics, Biometry, and Epidemiology, University Hospital Essen, University of Duisburg-Essen, Essen, Germany

PG and CH contributed equally as senior authors.

✉ Conrad-Amadeus Voltin, M.D

[conrad-amadeus.voltin@uk-koeln.de](mailto:conrad-amadeus.voltin@uk-koeln.de)

**
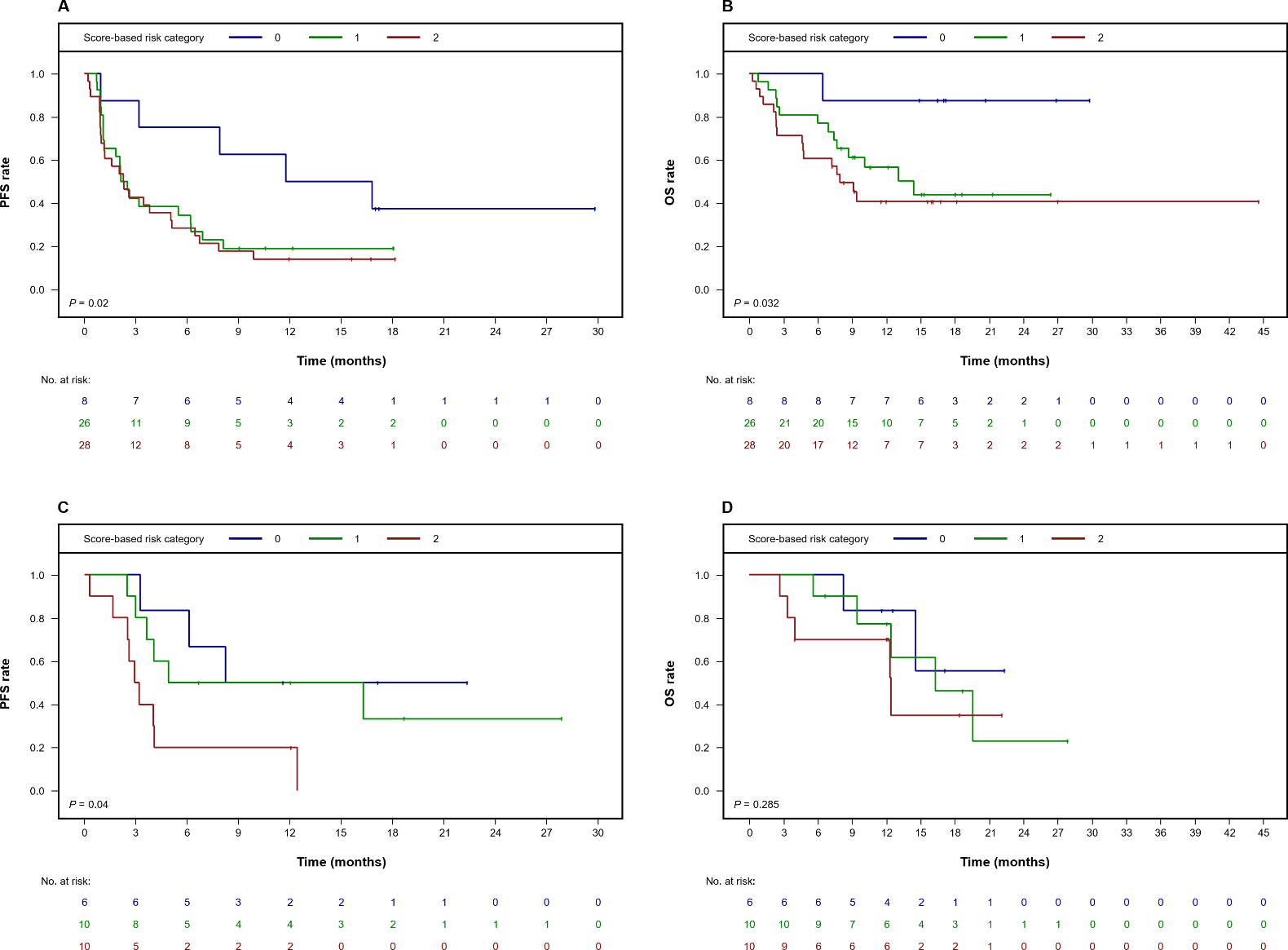
Supplementary Figure 1** Role of MTV and extra-nodal disease within the two treatment subgroups. Kaplan-Meier curves for PFS and OS in patients who received tisagenlecleucel (**A**, **B**) or axicabtagene ciloleucel (**C**, **D**) according to risk score. MTV, metabolic tumor volume; OS, overall survival; PFS, progression-free survival
